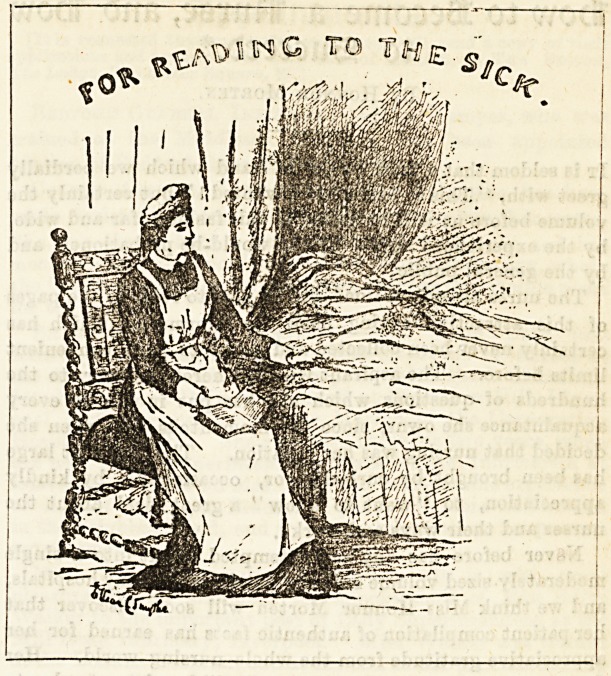# The Hospital Nursing Supplement

**Published:** 1892-10-01

**Authors:** 


### "The Hospital" Nursing Mirror.

Being the Extra Nursing Supplement of "The Hospital" Newspaper.

Contributions for this Supplement should be addressed to the Editor, The Hospital, 140, Strand, London, W.C., and should have the word "Nursing" plainly written in left-hand top corner of the envelope.

### En Passant.

"NURSING NOTES" AND ITS CHOLERA NUMBER. — The October issue of *Nursing Notes* will be welcomed by those who wish for accurate and unsensational teaching on the subject which engrosses so much present attention. Dr. Duncan's personal knowledge of cholera makes his lecture a valuable one, and the articles by Dr. Humphreys and Mr. G. M. W. Fenn will be appreciated by all nurses who want reliable instruction.

DERRY CITY AND COUNTY INFIRMARY.—The Very Rev. Dean Smyly made an astounding statement at the monthly meeting of the governors of this infirmary. He said, "From what we see of the management of the Board of Guardians, I believe they would starve them (the nurses) as well as they do the paupers," and he added, when cautioned, that he made the statement "on purpose." It will interest the readers of The Hospital to hear more of this.

DUNDEE ROYAL INFIRMARY.—At the quarterly Court of Governors it was decided to accept the tenders for the new Nurses' Home, and also to add another steam heater for the more satisfactory warming of the infirmary. The Matron of the Convalescent Home (Miss Ferguson) is leaving to be married, and is to be succeeded by Miss Bowman, a member of the nursing staff. The appointment has given much satisfaction. The Home is situated within a few minutes' walk of Barnhill Railway Station.

QUALITY IN QUARANTINE. — When travelling in any European country, save and except England, a certain period of quarantine is likely to fall to everyone's lot just now. These experiences are more varied than popular, but they have the merit of impartiality, and affect both prince and peasant. The King of Greece has been visiting his sister, and when he left her he had a prospect of five days' quarantine before re-entering Athens. However, the days spent on board the royal yacht need not be otherwise than pleasant ones.

CIGARETTES AND CYLINDERS.—In spite of much wise talk and many arguments for and against the habit of smoking, little boys continue to stroll about behind very large cigars, whilst big men content themselves with the modest cigarette. Visitors to the Laundry Exhibition lately held in Holborn were somewhat surprised to have the intricacies of the new inventions explained to them between whiffs of tobacco. Gentlemen were probably indifferent in the matter, but ladies did not appear to enjoy the cloudy atmosphere which prevailed in the Central Hall during the afternoons of this useful exhibition.

MACCLESFIELD COTTAGE HOSPITAL AND DISTRICT NURSING.—At the annual meeting of the trustees, committee, and subscribers, the Cottage Hospital was reported to be doing most satisfactory work, which is well supplemented by soup kitchen, Christmas treats, &c., the funds for which are supplied by independent contributions. These things are not deducted from the funds of the little hospital, for which more subscriptions are urgently needed. The District Nursing Association in the same town reports 909 visits paid by the nurse engaged five months before. This is excellent progress, and it is encouraging to read of the sick poor being taught something of ventilation, cookery for invalids, &c., during the calls of their valued Nurse Stuart.

ON ACTIVE SERVICE.—Besides Mrs. Watson, whom we have already reported as receiving the Royal Red Cross, we have the pleasant task of mentioning Miss Loch and Sister Luckfold, who have also been presented with the same honourable and distinctive decoration. They had previously received the Hazara Expedition medal for active service. We are always particularly pleased to receive tidings of honours conferred on trained and experienced nurses, when they have gone through the glorious but trying moments described in the two words "active service."

BAZAARS AND BENEVOLENCE.—A bazaar was held at Hockley early in September to raise funds for providing a district nurse The arrangements were most satisfactorily carried out under the supervision of Mrs. Tawke at Whitbreads, and various entertainments added to the attractions of the fête. There was a large assortment of useful articles at reasonable prices, and Mrs. Tawke provided a most liberal supply of refreshments in her garden. Another bazaar this month was held by children at Alfreton Vicarage in aid of the Trained Nurse Fund, and the young people were encouraged by a satisfactory attendance of visitors and purchasers.

ENGLISH INVALIDS AT SAN REMO.—People who are planning to spend the winter in North Italy will rejoice to know that an Institute for Trained English Nurses is to be opened this season at San Remo. The Lady Superintendent, Miss Bryant, was trained at the Children's Hospital, Pendlebury, and at St. Mary's, Paddington, and afterwards, held the post of Superintendent of Nurses at the Civil Hospital, Gibraltar. Miss Bryant has also had valuable experience in private nursing. and having spent the last two winters in San Remo, she is well able to judge of the urgent need for good English nurses, and she hopes to supply this want at 19, Via Vittorio Emanuele, San Remo.

LITTLEBOROUGH DISTRICT NURSING ASSOCIATION has published its first annual report, and this certainly testifies to able management, for there is a balance in hand as well as a good record of work. The honorary collectors well deserve the public thanks which the committee have cordially given them, for "it is through their exertions that the quarterly subscriptions of one shilling mount up to the good sum of twenty-five pounds." This is a practical way of increasing the fund, and also of maintaining a widely-spread interest in its progress. The rules appear reasonable ones, and all friction or doubt as to proceedings are certainly set at rest by the first, which reads thus: "Only such cases will be undertaken as are under the care of a medical man, whose instructions must be implicitly followed."

CLIFTON, BRISTOL, NURSES' CO-OPERATION has been formed, in the words of the prospectus, "To supply private nurses, thoroughly trained and certificated, to practitioners and the public, through the agency of the nurses, who have combined for their mutual benefit and welfare." Each nurse receives the full amount of her earnings, less 10 per cent., which will go to defray the yearly working expenses of the Co-operation. The Lady Superintendent was for thirteen years head of the Bristol Nurses' Institute, and must, therefore, possess valuable experience of the requirements both of nurses and the public at large. The cordiality with which those who have worked with her write of Miss Rogers' kind consideration for their comfort is a pleasant omen for her success in her new scheme. She has taken 14, Westbourne Place, Clifton, where she can accommodate the nurses between their cases.

### Lectures for Asylum Attendants.

By William Harding, M.B.

(*Continued from page* clxxxii, *Vol. XII.*)

### III.—CLEANLINESS.

To make a good nurse, much more is requisite than habits of cleanliness and order, but no nurse can be good without them. She cannot be held responsible for the character of the furniture, nor for the nature or taste of the decorations in her ward; but upon her rests the more important duty of seeing that everything is scrupulously clean and sweet. If she herself be untidy and slovenly in appearance when about her work, it is probable that that work will be performed in but a slip-shod manner. The example she will set to the patients will be a bad one, and her influence over those who have sufficient reason to notice these things will be small. She will find that many keen and by no means friendly critics are about her, who are ready to pass judgment upon her work and behaviour without being burdened with any scruples as to the plain and often scathing terms they employ.

If one finds the window ledges and corners of a ward littered with dust, and the backs of the ornaments wearing coverings of a similar nature, it is more than probable that the store-rooms are untidy, the boot-room higgledy-piggledy, the medicine chest in disorder, and the lavatories ill kept. As much pride should be taken in these places as in the brightest corner of the day-room, and they should always be ready for inspection. The nurse who is attentive to matters which do not come under the casual observer's eye, rarely allows her patients to be neglected or to appear untidy and unkempt.

It is perhaps in the care of her dormitory that the industrious nurse, with a love of order, is seen to best advantage. The sleeping rooms ought to be one of the pleasantest features of an asylum: bright and sweet, with white and stainless linen, spotless floor, and well-trimmed beds. A little attention to the position of the bedsteads, and the turning down of the sheets so as to keep them uniform, makes a wonderful difference in the appearance of the dormitory. The work, too, must be thorough. A clean upper sheet covering foul blankets or a dirty mattress; a pillow with a soiled side carefully turned down out of sight; the bedstead with an accumulation of dust deneath it—these are the things which vex a charge nurse's soul. Special attention is required in single rooms for dirty cases. Even where a bed is made on the floor with strong rugs, there is no reason why it should not be tidy, and the rugs, though strong, should be clean. We have already spoken about the care requisite in scrubbing out dirty rooms.

I speak with all deference, but I think that, as a rule, the dormitories are better kept in male than in female wards. The order is more manifest, and the beds are generally better made. Probably the more soldier-like discipline and the greater physical strength are the reasons for this difference.

The state of the patients' clothing should be a matter for constant observation. It will not do to rest content with changing at stated times. Active and intelligent interest is needed, as well as attention to a routine duty. Patients who are in the habit of hoarding up rubbish, rags, and scraps of food should be overhauled daily. A close watch should be kept to see that no food is carried from the dining-room to the day-room. Sufficient time should be allowed for meals, but no food should be taken from the table, and for important reasons other than those of cleanliness. Dirty habits at meal times, such as smearing the head with fat, &c., should be looked after.

Every individual, when it is possible, should have a weekly bath; in all cases for the sake of the individual's own health, and in some for the sake of the comfort of those who are compelled to associate with him. It seems a very simple thing to say that lunatics should be kept clean, but in practice it is not always so easily carried out. Many patients, of course, are extremely cleanly, and can attend to themselves, but unfortunately this class is in the great minority. In every ward there are persons who would avoid the weekly bath if they could, and many are the complaints and manœuvres to effect that end. Very dirty cases will often require bathing several times daily, and even then it will be difficult to keep them sweet. Some patients, who perspire freely and are very stout, are liable to have their skin irritated and chafed, especially in the folds, as under the breast and in the groin. These will require extra attention. The feet of others will give trouble to keep them sweet and prevent them chafing.

A point of great importance, and more particularly amongst those unable to attend to themselves, is the cleanliness of the hands. In some cases, undoubtedly, diarrhœas are caused, and parasites gain entrance to the intestinal canal, owing to the dirty habit of eating with unwashed hands and foul finger nails. In dirty cases especially, the nails should be cut short and the hands carefully cleansed before each meal. I am sure that if this were regularly attended to there would be fewer cases of diarrhœa among the very demented and dirty. It is, I grant, difficult in a large ward to carry out properly, but none the less, it should be done.

The condition of the teeth and gums, too, should not be forgotten. In some acute cases and in melancholics this is of importance, and ought to be seen to as part of the routine duty of the day.

In the admission ward, the condition of the heads of newly received cases is often a source of trouble, and even of anxiety to a careful nurse. Some cases are so very full of vermin that it is necessary to cut the hair short, but such extreme measures are seldom required. Patience, perseverance, and the small-toothed comb will do much with the occasional assistance of a little vinegar or other application to loosen the nits. Apart from these importations from without, it is a disgraceful thing to find a dirty head in an asylum. I know that some of the insane appear to furnish extremely favourable pastures for these vermin and that they seem to spring up by magic, but there is no excuse for their presence. The daily combing and dressing of the heads of those unable to look after themselves ought to afford a sufficient safeguard. The presence of dirty heads means simply that some nurse has shamefully neglected her duties. It is very seldom indeed that a flea is seen in an asylum.

### Examination Questions.

Answers to be sent in by October 29th, 1892.

*Prize: A Book on Nursing.*

I.—In what ways do sick children differ from adults, and what principles should be remembered in the Nursing, Feeding, Clothing, and Bathing of Infants?

Each answer must be accompanied by writer's name and address, must be short and concise, written on one side of the paper only, and addressed "*Nursing*" Editor of The Hospital.

### Wants and Workers.

*M.E.Beacall, 13, Ridgway Place, Wimbledon*, will be very grateful if any reader of The Hospital will kindly give her information as to how she can procure patterns for dressing "doll nurses" for a group in a doll show to be held early in December for the benefit of the G.F.S. Sick Fund.

*Nurse May* will be very thankful for information about Denver City, Colorado. Are there any homes where an invalid and his wife could be received at moderate terms?

*Sister Lucie* will be glad of old linen and second-hand clothing for the sick poor. Address *Sister Lucie, Littleborough District Nursing Association.*

Wanted, a home for a cripple boy (10), where he can be educated. Mother dead, father respectable tradesman. Permanent invalid.—*Miss J. Russell, 9, St. Margaret's Road, St. Leonards-on Sea.*

A trained nurse (widow) is anxious to find employment for son, aged 19. He enjoys good health, but has a stiff knee, result of an accident. Accustomed to office work. Educated at The Oratory Middle School. *Address Nurse Kerr, 38, Ladbroke Road, Notting Hill.*

### Neat Beds.

Bed-making is an essential part of nursing work, as so much of a patient's comfort depends on the method and the hour when this is done for him. It is not only at the time when the regular morning work includes the changing of sheets that a sick man's bed needs attention. It has to be looked to after each meal in case chance crumbs have gone astray, and it must be thoroughly re-made in the evening to give the patient every chance of a quiet night. In private nursing it is well to let the convenience of the household, as well as the fancy of the patient, decide the precise moment when the bed shall be done, but it is always best to observe some regularity in the matter. At first a sick person often complains of the "unnecessary" second making of his bed, but after a day or two we generally find him looking forward somewhat eagerly to the moment when nurse comes to "settle him down" again. Experience shows him the advantages of the plan, and he values the increase of comfort.

When a patient is either very ill or very seriously injured, of course many precautions must be taken to avoid all possibility of risk, and perhaps for many days the bed can only be attacked to a very limited extent. But naturally the majority of hospital patients are in neither of these extreme conditions, and are quite prepared to enjoy the break in the monotony of their long days. In the hands of a skilful nurse the performance is quite an interesting one, and her deftness and quiet rapidity cause the invalid to ejaculate incredulously, "Is it really finished, nurse?" The bed of a person who is very weak should always be made in silence, although the temptation to talk, when two nurses are engaged upon it, is probably a strong one. But there is no doubt that conversation is trying to the patient, who finds that one thing at a time is all he can bear patiently in his present condition. And if talking *to* him is bad, talking *about* him is far worse, and an attendant possessed of that tact which goes so far to make a nurse sympathetic, will seldom err in this way. Still we do, unfortunately, constantly encounter women who are misguided enough to think that a running conversation, made up of unnecessary items, is essential to her charge's well-being. A cheerful and encouraging word is quite as much as he cares for, and he is often driven to irritability by well meant but injudicious attempts at "raising his spirits," when he is mainly concerned with the temporary feebleness of his body!

In spite of the splints, sand bags, "cradles," &c., which distort into marvellous shapes the bedclothes in a surgical ward, the general effect gives an impression of orderliness. A man or woman who is attached to a splint, having a bad bone injury, is comparatively still in bed, and a person who has met with a serious accident is often very quiet indeed, and his coverings remain in perfect order.

When we get into medical wards, however, we find a different state of things. The feverish patient turns and tosses, and tries in vain to find a comfortable position; and as he moves from side to side the clothes are thrown hither and thither, and "I can't keep his bed tidy!" exclaims nurse in despair. A high temperature or excessive pain alike make stillness absolute torture to the sufferer, and it should never be demanded from him. What cares he for the neat appearance of a bed which gives no rest to his pain-racked frame? Then let us, if we cannot give present ease, at least avoid adding to existing trials by the foolish injunction to "keep the clothes tidy." It is as inconsiderate as it is useless, and, after all, much as we may admire an orderly ward, we must admit that the comfort of the patient should rank higher than the vanity, however pardonable, of the nurse.

Pillows and counterpanes, blankets and sheets, must be put in order, and straightened with admirable precision, but patients must never be blamed for their failure to maintain this condition of things. The days passed in bed are wearily dull very often, and the burden of the endurance of suffering is a weight which should never be made heavier by the needless addition of irritating remonstrances.

### THE NETTLE.

If we face our difficulties in life boldly we shall find them less painful and much easier to bear than when we dread them, and are always trying how we can get rid of them. "Grasp your nettle," says the old proverb, and the truth of it comes home to everyone who, while gathering wild flowers, has brushed against the harmless-looking weed. We at once feel a hot burning itching which torments us for hours, and nothing but patience and a dock-leaf make it bearable; whereas if we had had courage to clutch the plant and throw it on one side to clear the way to the blossoms we coveted, we should have had very little inconvenience. A good many nettles are found in everybody's path as we grow older, which spoil our comfort terribly. There are the ordinary trials of discontent and ill-temper in ourselves and others, failure in our attempts to get on in life, little misunderstandings with friends. These want looking at boldly and handling with a will, and we shall only feel a passing throb from having met with them. And if we are stung by an unkind word, "the soft answer which turneth away wrath" is the dock-leaf to put on the place. But there is a nettle which stings us sometimes when we are not prepared for it, and that is sickness. It comes at times and seasons when we least expect it, and so weakens body and mind that we are powerless to make any efforts against it. Those who have been in the habit of grasping their nettle in health are the best off now, for they bear their throbs and agonies with fortitude, and do not by their impatience chafe their minds and bodies and make them worse. Now it is well known that wherever a nettle grows there is always a dock-weed near which, if gathered and put on the sting, gives relief, and the leaves we should fly to in suffering are gentleness and patience, fortitude, cheerfulness and love.

We need not seek far to find them, they are all on the "Stem of Jesse," that beautiful "Plant" which was sent for the healing of the nations—our Lord Jesus Christ. But as the dock-leaf is of no use to ease our pain without gathering it, though we may look at it for hours, so unless we go to Christ and ask for these gifts we shall continue in our trouble. That our Lord will not only help us to bear our agonies, but take them away if we pray to Him earnestly and in a faithful loving spirit is certain. He who when on earth healed the lepers and sent away the maimed, the deaf, the halt, the blind perfectly cured, when they believed on Him and came to Him, will do the same now for us. His hand is not shortened that it cannot save now, and having borne in His own body the ills which flesh is heir to, He is touched by our infirmities, and with compassionate love removes our sickness, or if it should be good for us to be afflicted, will give us such comfort and patience and hope that we shall rejoice that day by day we grow liker to our dear Lord.

### How to Become a Nurse, and How to Succeed.*

By Honnor Morten.

It is seldom that a book comes to hand which we cordially greet with, "This is just what I wanted!" but certainly the volume before us will be hailed in this fashion, far and wide, by the experienced nurse, by the would-be probationer, and by the general public.

The nurse already trained will be glad to refer to the pages of this attractive-looking book for information which has certainly never been collected and put into such convenient limits before. The aspirant will find herein answers to the hundreds of questions which she has put in vain to every acquaintance she owns, since the momentous day when she decided that nursing was her vocation. The world at large has been brought by curiosity, or, occasionally, by kindly appreciation, to "want to know" a great deal about the nurses and their ways and works.

Never before has any one attempted to put into a single moderately-sized volume so many details of so many hospitals, and we think Miss Honnor Morten will soon discover that her patient compilation of authentic facts has earned for her appreciative gratitude from the whole nursing world. Her first chapter, headed "Application," will be of great value to the young girl whose whole soul is set upon "being a nurse," but whose ideas of how to approach the desired goal are of the vaguest description. A careful perusal of this Chapter I. will certainly make her a wiser woman, and it will help her to make clear to herself and her friends the exact steps up which she aspires to climb.

"Probation" is the title of an excellent chapter which contains many valuable hints which the writer gives delicately under the guise of "hospital etiquette," a poetic rendering of "good and becoming manners." Any inculcation of these is much to the point, and cannot be too highly commended.

Certificates, and the way in which they are earned, ends the first part of the subject, and chapter IV. introduces us first to London hospitals, and afterwards to provincial, Scotch, and Irish training schools. The hours on duty, the pay, holidays, &c., of probationers, are given in most cases, besides other valuable information.

A chapter is devoted to the important subject of Private Nursing, which is very well dealt with, and will be of great value to those who incline to follow this branch of work.

District nursing has received from Miss Honnor Morten the attention which it needs and deserves, and the details she offers will be of use to many who desire to become "Queen's Nurses" She gives a quotation from Miss Nightingale's letter, written to Lady Rosebery at the starting of the Jubilee Institute, which contains the suggestive words "besides a nurse, she must be a sanitary missionary," which was certainly a most happy description of one part of the district nurses' duties. The chapters devoted to eminent nurses naturally begin with one whose name is a household word—and her portrait with equal propriety forms the frontispiece of the book; a sketch of Miss Florence Nightingale's life and work will certainly add to the attractions of "How to Become a Nurse" in the eyes of many readers.

Specimens of examination questions, lists of societies and guilds, and the names and prices of books likely to be of help to studious nurses, are all to be found in this handy volume, which we most cordially commend to The Hospital readers.

* "How to Become a Nurse, and How to Succeed," by Honnor Morten, price 2s. 6d., published by The Scientific Press, 140, Strand.

### Hints for Nurses.

### WARM DRESSES.

The colder and shorter days warn us that autumn is come, and winter will soon follow; and, acknowledging the unwelcome truth, our thoughts are forced to turn to warm clothes and the woollen materials which are necessary for about eight out of the twelve months in our English climate. We have before us some charming patterns manufactured by Egerton Burnet, Wellington, Somerset, and the serges especially commend themselves to us as fulfilling a nurse's requirements. Besides their uniform dresses, nurses seldom care to be burdened with a great variety of costumes, as they are apt to get old-fashioned long before they get worn out by holiday service. Good serges never look antiquated, and seldom have a shabby air, and therefore it is worth while to get one of the "Royal Serges," which Messrs. Burnet make in such variety, "plain" and "fancy," narrow and wide, and at all prices.

The Plinlimmon wrap shawls are also much to our taste, and one of them will prove a permanent comfort to a private nurse, whose sudden and frequent journeys will assuredly be less trying at cold seasons, if she supplies herself with this inexpensive luxury. The Park Lane skirtings are very good too, and a stout petticoat, warm and pretty withal, is quite as essential as a stylish dress. These skirts are made in every colour and design, and with such a choice before her, no nurse will have a difficulty in selecting patterns to her taste.

### CARDS FOR DISTRICT NURSES.

Miss Katherine Twining has sent us a specimen of a report-card which is used by her district nurses at Plaistow, and we feel sure that it will commend itself to our readers. It is a grey correspondence-card of the same size, and with perforations also copied from the Post Office ones. The outside is blank: on the inside we find printed the simple headings under which a nurse would naturally note her observations for the doctor, and it is thus made easy for her to leave an accurate record of facts on this card, gummed up and addressed to the medical man. We all know how unsatisfactory it is to trust verbal messages or unenclosed notes at a cottage, and the present seems a plan to please both doctors and nurses.

### SOFT WATER.

At the Laundry Exhibition recently held at Central Hall, Holborn, we saw an excellent patent water-softening plant, designed by Messrs. Doulton, of Lambeth, which could be conveniently used in private houses as well as in institutions. The saving in labour as well as in soap when soft water is obtainable should commend this invention to all housekeepers. The powder used for converting hard water into soft, can be obtained in small quantities, scented or unscented, and we strongly recommend the latter to all nurses who wish to keep their hands normally soft and sound amidst the drawbacks of their daily work. As we have pointed out elsewhere, it lies with the nurse herself to keep her own skin in good condition, but this result will be easily accomplished if a small pinch of Doulton's powder is put into the ewer some twelve hours before the water is required. Care must be taken to adhere strictly to the directions given as to quantity, for too much powder put in at one time neutralises the desired effect.

### Presentation.

Miss Garnett Clarke, on leaving Nailsea after three years' work amongst the sick poor, was presented with a handsome pair of silver table lamps and a silver-plated soup tureen and ladle, with many expressions of goodwill from her friends and well-wishers. Miss Clarke is about to open a Private Convalescent Home for Paying Patients at Ellastone, Walton Bay, Clevedon, Somerset.

### Everybody's Opinion.

*[Correspondence on all subjects is invited, but we cannot in any way be responsible for the opinions expressed by our correspondents. No communications can be entertained if the name and address of the correspondent is not given, or unless one side of the paper only be written on.]*

### ODDFELLOWS AND DISTRICT NURSING.

"Sister Lucie" writes: Allow me to thank you most sincerely for so kindly inserting amongst "Wants and Workers" two appeals for homes: one for a girl, and the other for a helpless elderly lady. I am glad to tell you that I have, through the medium of The Hospital, found suitable homes for both, and I am most grateful for your assistance. I forward by this post the first annual report of the Littleboro' District Nursing Association; since it was printed the "Oddfellows" have had a demonstration, and have handed over £7 10s. to the Association. This donation was most acceptable, as well as unexpected, for the Oddfellows had not been asked to help; it was entirely their own kind thought.

### NURSES' CO-OPERATION AT CLIFTON.

"Nurse M. A. C." writes an excellent letter from Clifton, Bristol, telling us of the Nurses' Co-operation recently established there. We thank her for the information, to which we refer in another column. Nurse M. A. C. is one of the First Thousand, and we congratulate her on her good fortune in that respect.

### TRAINED NURSES CLUB.

"A Trained Nurse" says: It is proposed to hold a drawing-room sale of work in aid of the funds of this club early in December, and the Committee are very desirous of obtaining promises of contributions or other help. Useful or ornamental articles likely to sell will be received and acknowledged by Mrs. Nichol, 12, Buckingham Street.

### FOOD AND WAGES.

"An Experienced Nurse" writes: Dear Mr. Editor,—Can you tell me something about the Derry Infirmary. I read the account of a recent meeting of Governors when some one proposed that one of the nursing staff should be "always available" in case cholera arrived in Lough Foyle. Sir William Miller wisely declined an arrangement which would cripple the internal management. Of course nurses kept expressly for such emergencies would have to be paid by the same authority as provides the remuneration for special medical officers. But, Sir, I want to know if there is any truth in the Very Rev. Dean's assertions, because although most nurses are willing to take cholera work, they could not attempt to do it on insufficient rations.

### Notes and Queries.

### Answers.

*Cholera (Ellen).*—"Nursing Notes" for October will contain report of Dr. Duncan's lecture.

*Probationer (Jane).* You will get the information you desire in "How to Become a Nurse," by Honnor Morten, price 2s. 6d., Scientific Press, 140, Strand.

*Doll Nurses (M.E.B).*—The case containing dolls dressed in nursing uniforms is to be seen at The Hospital Office, 140, Strand.

*Continental Hospitals (Weymouth).*—If you wish to enter a German hospital, you will require to have a thorough knowledge of the language.

*Nerves (Joseph).*—You must give name and address if you wish your query answered.

*Trained (A Free Lance).*—(1) No, the title "trained" should only be adopted by nurses who have earned a two or three years' certificate in a general hospital.—(2) Call at R.N.P.F. office, 8, King Street, or write there to Secretary.

*Training (One Who Loves Her Work).*—"How to Become a Nurse," by Honnor Morten, will answer all your queries.

*Son (Nurse Kerr).*—Look in "Wants and Workers."

*A Future Pro. (B.).*—You could not have chosen a better infirmary than the one at Gloucester. The Matron has had a complete training and splendid subsequent experience. We wish you success.

### Appointments.

[It is requested that successful candidates will send a copy of their applications and testimonials, with date of election, to The Editor, The Lodge, Porchester Square, W.]

Bedford General Infirmary.—Miss Wemyss, who was trained at the Middlesex Hospital, has been appointed Matron of Bedford General Infirmary.

County Asylum, Lancaster.—Miss Valentine has been made Assistant Matron at the County Asylum, Lancaster. She was chosen from a large number of applicants, and has most excellent testimonials from the Wigan Infirmary, where she has been a highly-valued nurse, holding responsible posts for ten years.

Bradford Fever Hospital.—Mrs. Kate Daly (*née* Kearney) has been appointed Superintendent of nurses at the Bradford Fever Hospital, a post which she is eminently fitted for. Mrs. Daly was trained at Brownlow Hill, Liverpool, and subsequently became Matron of the Corporation Hospital at Bootle. She remained there for two years, when she was made Superintendent-Nurse of the Infirmaries at Leavesden. Later on Mrs. Daly had considerable experience of district nursing in Bradford; she is therefore well known in the neighbourhood, and we congratulate her on her present appointment.

Mill Road Infirmary, Liverpool.—It is with great pleasure that we announce the appointment of Miss Edith Walker to the important post of Lady Superintendent at the Mill Road Infirmary, Liverpool. Miss Walker was trained at the London Hospital, and at the end of her probationership was made a Sister, and has acted as "Matron's Assistant" for a period of nine years, so that her connection with the nursing staff has covered altogether eleven years. Miss Walker has had unusual opportunities for acquiring experience in the management and organisation of an exceptionally large and successful nursing school, and we congratulate the Committee of the Mill Road Infirmary on their good fortune in securing her services.

London Hospital.—Miss Fynes-Clinton, for many years Sister at the London Hospital, has been made Assistant Matron in place of Miss Walker.

### An Interesting Presentation.

The Governors of the Mill Road Infirmary, Liverpool, have certainly proved themselves to be most sagacious gentlemen by their action in the selection of Miss Edith Walker as Lady Superintendent for their new nursing home. They have decided on organising a scheme of trained nursing in their new buildings and have spared themselves no trouble in the matter. The formal notice that "canvassing will be deemed a disqualication" was literally adhered to, and we certainly congratulate the committee on this honourable resolution. They not only saw the selected candidates officially at Liverpool, but a deputation of three experienced gentlemen was formed to visit these ladies in their own hospitals. Those who know the London Hospital will realise the good impression it made on the visitors and their appreciation of the orderly condition of that institution. We may venture to predict a brilliant future for the new nursing school, and we feel sure that the Medical Superintendent will find a most loyal coadjutor in the lady who has done such admirable, unobtrusive and conscientious work for the last eleven years. The Nursing Staff have presented Miss Walker with a beautiful silver tea service, and her popularity in all departments is proved by a gift from the servants and porters of a Morocco case containing a pair of elegantly chased fruit spoons. If good wishes and cordial congratulations ensure prosperity, Miss Walker's future career will be an enviable one.

### Where to Go.

Ten Lectures on Shakespeare, by Mr. F. S. Boas, M.A., Balliol College, will be given in Morley Memorial College, Waterloo Bridge Road, at eight o'clock on Friday evenings. First lecture, October 7th. Particulars may be obtained from Miss Cons, hon. sec., Morley Memorial College.

Science Lectures at same hall on Tuesday evenings. Hon. Manager, Miss Cons, Morley Memorial College.

### The Result of a Chill.

(*Continued from page* clxxxvi, *Vol. XII.*)

### MONITION OF TWO KINDS OF FEVER.

The cleave was filled with golden vapour, when the visitor at Hollacombe threw wide the lattice and looked out at the fading hues of sunrise, and the rich burning brown of the moor. The wind had raged itself to sheer weariness, and had fallen asleep at daybreak with a few faint sighs. Not a frond of fern nor leaf of a beech moved, and in the vast mysterious silence of the hills came no break but the cry of a grey heron as it rose from the swollen stream. The visitor descended, and heard the girls in the kitchen, while in the court behind the house a whistling boy was beginning to hew wood with a curved chopper. The thought of a moorland view inspired him to ascend the western hill, and he went up, through wet scented heather and clitters of granite rock, to a cairn-like pile of stones on the crest. There he sat down, and began to picture Sybil as she sat in the chimney-corner. He had seen many fair women in his time in many countries, and he was, to his thinking, something of a connoisseur of feminine loveliness. As to the one particular charm of the elder sister he could not decide just then, but he confessed she had a face that gave him infinite pleasure. It was first of all an intellectual countenance, with a frank kindly look that met his own with a suggestion of rapidly-conceived friendship. There was little doubt that his hostess had appealed to his imagination upon first sight, and the fact that the contour of her features were foremost in his waking perceptions brought conviction that she had already no common interest for him. He sat till a shiver and a sneeze warned him that a chill was upon him, the probable result of yesterday's soaking, and the want of a complete change of clothing. He went down the hill, humming abstractedly. Sybil met him in the garden, exchanged a good morning, and said breakfast was ready.

"I must apologise again for our fare," she said. "Your choice lies between porridge and eggs; we gave you eggs last night. The butcher comes but once a week, and we have no joint in the house."

The stranger mentally avowed that he could have feasted upon a dry loaf in such company, and he was almost minded to say so. But he told Sybil that he was too much obliged for her kindness to object to any sort of food she might place before him, and as for porridge, it was one of his likings, born of Scottish ancestry on his mother's side.

After breakfast he took up Sybil's picture once more, and stood it in a good light near the window.

"Will you sell this?" he said to her.

"With pleasure, I paint for sale," she answered; "it wants varnishing though."

"Never mind that. Tell me the price, and if you will send it to this address I may be able to sell it." He wrote in pencil, "F. Lowther, picture dealer, Bury Street, Pimlico," on the back of the canvas.

"I shall be glad to get six guineas for it," said Sybil.

"It ought to bring more, and I think it will. Leave the price to me, now that I know what value you place upon it."

Sybil consented in perfect confidence, and he entered her name and address in a memorandum book.

"And now," he said, "permit me to thank you very deeply for your hospitality. My holiday nears its end, and in three days I shall be in very different surroundings, where the recollection of your happy valley will fill me with still greater envy for your placid life. Good-bye. I shall find my way without any trouble, thanks to your directions."

He swung his knapsack on to his shoulders, shook the girls' hands, and went down the garden swinging his stick. From the deep window Sybil watched his figure silhouetted on the brow of the hill, and when it disappeared she sat down, with her hands on her lap, and looked at the picture of Kestor. For over six months she had not exchanged an idea with a man of education and taste, and the brief intercourse with the interesting stranger, who had broken in so unexpectedly upon the monotony of her life, had been a time of mental stimulus and enjoyment. His face, his deep rich voice, his air of culture had impressed her strangely. There was something so magnetic and intensely alluring in his personality that more than a tinge of disappointment for the speedy termination of his visit entered into her reflections. During that day she acknowledged that if she had met him in society his air and tone would have stamped him as a polished man of the world; but the somewhat romantic circumstances of their meeting invested his sociability and kindliness of disposition with peculiar attractions. Both the sisters admitted that no more charming guest than the wanderer could have been fortuitously introduced to Hollacombe. They speculated upon the probability of meeting him again, but such an event seemed vague in the extreme. It was, perhaps, odd that neither should at first suspect their guest to be F. Lowther, of Pimlico. Yet that was the name he had written on the canvas, and he had given no other. Late on the night of his leave-taking it flashed simultaneously upon their ingenuous perceptions that the errant stranger was no other than a London picture dealer. This was no rational cause for immediately changing their opinion of his courtesy and refinement, but the idea was so ludicrous that they looked at each other and laughed.

"If he is a tradesman, he is certainly the most intellectual I have ever seen," observed Sybil, whose estimate of the mercantile class was largely prejudiced by her mother's recent marriage with the distiller.

Hilda argued that there are tradesmen who are shoppy and tradesmen who are not of the shop, and from this she proceeded to cite cases of gentlemanlike shopkeepers.

"After all, what does it matter?" said Sybil.

Nevertheless, on the morrow it mattered more to her inner self than she chose to avow to her sister. For it disconcerted her to think that the easy and charming air of the stranger was only the outcome of long years of suave haggling with aristocratic customers in a back street of Pimlico. She had begun to image him working in a fine studio, surrounded by the works of his brush. Had he not spoken of pictures like a painter? Supposing him to be only a dealer, would not his criticisms have hinged mainly on the question of marketable value?

A week passed after the despatch of the painting, and no news came from Pimlico. Hilda, always the more practical, declared that Mr. Lowther ought to have intimated the safe arrival of the package. To this Sybil said simply, "How do we know his name is Lowther?" and took up her palette.

"Well, dear, we don't," returned Hilda, who perceived that any suggestion of the visitor's negligence was apt to pique her sister. "His name may be Orlando de Crespigny for aught we can tell, though Lowther is not altogether plebeian. It might be Brown. I should like, however, to inform you that we shall be in desperate straits soon if some money doesn't come."

"How much have we in hand?" asked Sybil, squeezing yellow ochre out of a tube.

"Thirty-five shillings and a penny, and the rent is due."

*(To be continued.)*

## Figures and Tables

**Figure f1:**